# Changes in estimated glomerular filtration rate before and after the first visit for atrial fibrillation

**DOI:** 10.1186/s12882-023-03449-y

**Published:** 2024-01-03

**Authors:** Ryobun Yasuoka, Hiroyuki Sakurane, Mana Okune, Motohide Tanaka, Tomoya Nagano, Masahiro Maruyama, Gaku Nakazawa, Takashi Kurita

**Affiliations:** 1 Division of Cardiology, Department of Medicine, Faculty of Medicine, Kindai University, 377-2 Ohnohigashi, 589-8511 Osakasayama, Osaka Japan; 2 Division of Cardiology, Kiwa Hospital, 18-1 Kisikami, 648-0085 Hashimoto, Wakayama, Japan

**Keywords:** Atrial fibrillation, Worsening renal function, Inflection-point

## Abstract

**Background:**

Although the development of atrial fibrillation (AF) and the progression of chronic kidney disease are known to be interrelated, it remains unclear when and how renal function changes during the clinical course of AF.

**Methods:**

This study retrospectively enrolled 131 patients who were able to collect data on estimated glomerular filtration rate (eGFR) at least five times during the 500 days before and 500 days after the first visit (baseline) of new-onset AF, respectively. To investigate the temporal relationship between the development of AF and the beginning of worsening renal function (WRF), a piecewise regression model was applied to the eGFR time series data. The time point at which the slopes of the two regression lines changed (inflection -point), the slope before and after the inflection-point (β1 and β2, respectively), and the difference in slope (Δβ) were estimated. The presence of WRF was defined as having the inflection-point at which both Δβ and β2 were < − 0.0083 mL/min/1.73 m^2^/day (corresponding to 3.03 mL/min/1.73 m^2^/year), and the corresponding the inflection-point was defined as the beginning of WRF.

**Results:**

WRF was detected in 54 (41.2%) patients. The beginning of WRF were distributed at various times, but most frequently (23 of 54 patients) within 100 days before and after baseline. The presence of WRF was not associated with age, heart failure, or baseline eGFR, but was associated with positive β1 (odds ratio 30.5, 95% confidence interval 11.1–83.9, *P* < 0.01).

**Conclusion:**

In nearly half of AF patients with WRF, the beginning of WRF was observed within a few months before or after the first visit for AF. Patients with a positive eGFR slope before the onset of AF are more likely to develop WRF after the onset of AF, suggesting that potential kidney damage may be underlying.

## Introduction

Several investigators have reported that atrial fibrillation (AF) increases the risk of chronic kidney disease (CKD), and that CKD increases the risk of new-onset of AF [1–3]. It has been recognized that AF and CKD are closely interrelated, with either being a possible cause and result of the other. However, almost all interest has focused on changes in renal function after the development of AF [4–6]. We conducted a study to identify when and how renal function changes during the development of AF.

## Methods

### Study design and inclusion criteria

**Fig. 1:**
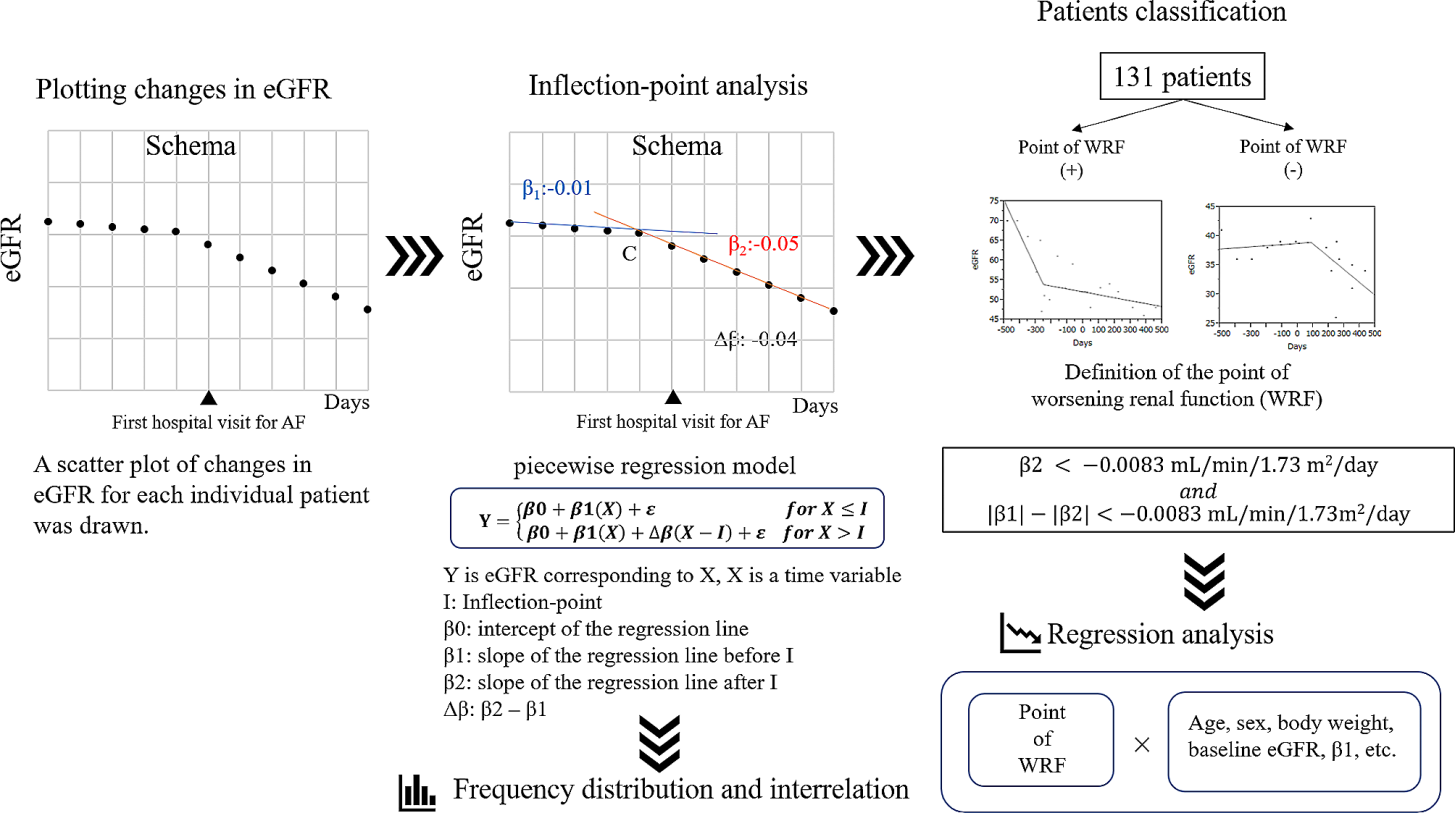
Flowchart of methods for analyzing changes in eGFR before and after the first visit for AF. *eGFR* estimated glomerular filtration rate

This was a single-center, retrospective study conducted at Kindai University Hospital from May 2011 to October 2018. Using clinical information from medical records, we identified and screened 546 outpatients with AF on anticoagulant therapy. Of all patients, those for whom data measuring renal function were obtained at least 5 times in each period over 500 days before and after the occurrence of AF were selected. The day of the first visit for the new-onset AF was defined as baseline (day 0). The enrollment criteria were set as follows: (i) documentation of newly diagnosed AF (presumed to have symptoms of AF within a few months prior to the time of baseline), (ii) estimated glomerular filtration rate (eGFR) of at least 15 ml/min/1.73 m^2^ at baseline, and (iii) there were less than three missing intervals of eGFR data (per 100-day interval) before and after baseline. A flowchart of the methods is shown in Fig. 1.

### Estimation of renal function

Renal function was estimated from Japanese equation for eGFR as follows: eGFR (unit: mL/min/1.73 m^2^) = 194 × serum creatinine ^− 1.094^ × age ^− 0.287^ (× 0.739 if female sex) [7]. The serum creatinine levels obtained at each visit were examined from the medical records. Because a fatal condition affects renal function, data within 60 days before death were excluded from the analysis. If blood sample data were not available on day 0, the eGFR on the day closest to day 0 (within 1 month) was substituted for the baseline data.

### Inflection-point detection and slope estimation in the eGFR

A scatter plot of changes in eGFR for each individual patient was drawn. A piecewise regression model was fitted to the scatter plot to identify the inflection-point, which represents the point of greatest change in eGFR during the follow-up period, and to estimate the slope before and after the inflection-point. The reason for using this statistical model was to examine the temporal relationship between the maximum change in eGFR and the onset of AF. If the maximal change in eGFR occurred close to the time of the first visit for AF, then it could be assessed as directly related to the development of AF. Conversely, if the maximal change in eGFR occurs far from the time of the first visit for AF, the association between the maximal change and the development of AF is weak. Piecewise regression models are statistical models in which two lines are joined at an unknown inflection-point. The model and parameters of the piecewise regression analysis are as follows.

$$Y = \left\{ {\begin{array}{*{20}{c}}{\beta 0 + \beta 1(X) + \varepsilon }\\{\beta 0 + \beta 1(X) + \Delta \beta (X - I) + \varepsilon \,\,\,\,\,}\end{array}} \right.\begin{array}{*{20}{c}}{for\,X\, \le \,I}\\{for\,X\, > \,I}\end{array}$$

$$ \varDelta \varvec{\beta }=\varvec{\beta }2-\varvec{\beta }1$$

*β*_0_: intercept of the regression line before the inflection-point.

*β*_1_: slope of the regression line before the inflection-point (units: mL/min/1.73 m^2^/day).

*β*_2_: slope of the regression line after the inflection-point (units: mL/min/1.73 m^2^/day).

Δ*β*: difference between the eGFR slope before and after the inflection-point (i.e., *β*_2_ minus *β*_1_).

where Y is the value of the eGFR corresponding to X, X is a time variable, and the parameter I is the time variable at the inflection-point. The parameter *ε* was assumed to be an independent, additive error with a mean of zero, constant variance, and a finite absolute moment of some order > 2 [8, 9]. The inflection-points were statistically detected by using a maximum likelihood, where the maximum probability of a change in slope occurs. Estimated inflection-points included those with subtle, clinically insignificant slope changes. The estimated inflection points were utilized for classification based on slope criteria before and after the inflection-point, as described later in the next section.

### Definition of worsening renal function

We divided the estimated statistical inflection-points into those with and without worsening renal function (WRF). WRF was defined as having an inflection-point with both Δβ and β2 of < − 0.0083 mL/min/1.73 m^2^/day, and the corresponding inflection-point was defined as the beginning of WRF. This criterion can be expressed by the following equation:

$${\rm{WRF}}\,{\rm{positive}}\,{\rm{criteria = }}\left\{ \begin{array}{c}{\rm{\beta 2}}\,{\rm{ < }}\, - {\rm{0}}{\rm{.0083}}\,{\rm{mL/min/1}}{\rm{.73}}\,{m^{\rm{2}}}{\rm{/day}}\\and\\\left| {{\rm{\beta 1}}} \right| - \left| {{\rm{\beta 2}}} \right|\, <- {\rm{0}}{\rm{.0083}}\,{\rm{mL/min/1}}{\rm{.73}}\,{m^{\rm{2}}}{\rm{/day}}\end{array} \right.$$

Cases in which renal function improves before the inflection point but declines thereafter, and the absolute values are approximately balanced, do not meet the criteria for WRF (i.e., are not defined as WRF).

This criterion is based on the fact that in some clinical trials, the rate of decline in eGFR is considered rapid if the rate exceeds 3 mL/min/1.73 m^2^/year (0.0083 mL/min/1.73 m^2^/day in daily calculations) [10, 11].

### Representative cases with and without WRF

**Fig. 2:**
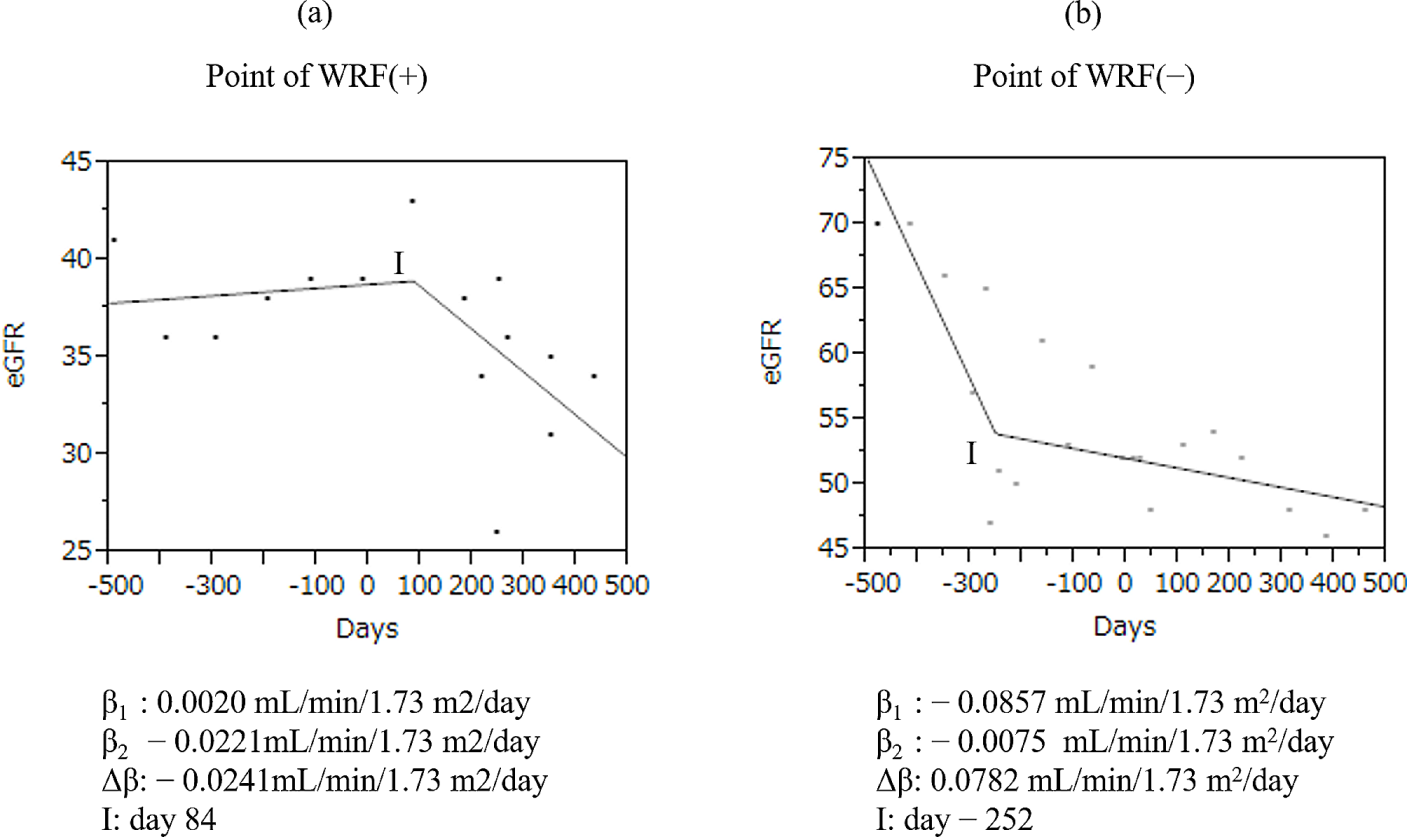
Scatterplots of the change in the eGFR in two representative cases. Scatterplot of a patient with the point of WRF (**a**). Scatterplot of a patient without the point of WRF (**b**). *C* the inflection-point, *β1* slope of the regression line before the inflection-point (units: mL/min/1.73 m^2^/day), *β2* slope of the regression line after the inflection-point (units: mL/min/1.73 m^2^/day), *Δβ* β2 – β1(units: mL/min/1.73 m^2^/day)

Figure 2a shows a case with WRF (day 84; β1 [0.0020 mL/min/1.73 m^2^/day]; β2 [− 0.0221 mL/min/1.73 m^2^/day]; Δβ [− 0.0241 mL/min/1.73 m^2^/day]). Figure 2b shows a case without WRF. The inflection-point was detected at day − 252, but did not meet the criteria for WRF, with Δβ of 0.0782 mL/min/1.73 m2/day (β1 [− 0.0857 mL/min/1.73 m^2^/day]; β2 [− 0.0075 mL/min/1.73 m^2^/day]).

### Clinical variables

Baseline clinical variables at the first visit for AF including age, sex, body weight, chronic heart failure (CHF), hypertension, diabetes mellitus, stroke, CHADS_2_ score [12], vascular disease, type of AF (paroxysmal or persistent AF), diuretics, angiotensin-converting enzyme inhibitors and/or angiotensin-receptor blockers (ARBs/ACE-Is), left atrial dimension (LAD), left ventricular ejection fraction (LVEF), baseline eGFR, and baseline CKD class (according to the Kidney Disease: Improving Global Outcomes [KDIGO] classification) were investigated [13].

### Statistical analysis

Continuous variables were expressed as the mean ± standard deviation or median and quartiles. Categorical variables were expressed as the number of patients and percentage. Univariate and multivariate regression analyses were performed to examine which clinical variables were related to WRF. We carried out multivariate regression analysis in an adjusted model including the following clinical variables at the first visit for AF that are generally recognized to be associated with CKD stage and/or CKD progression in patients with AF: age, [2, 14–16], sex [2, 3, 14, 15], body weight [6], CHF [2, 6, 14–17], elderly patients (> 75 years) [6, 14], hypertension [1, 3, 14], diabetes mellitus [1, 3, 14], stroke, CHADS_2_ score (≥ 2 points) [14, 15], vascular disease [2, 14, 17], type of AF [14, 16], diuretics [14, 16, 17], ACE-Is/ARBs [14, 16], LAD [16], LVEF [16], baseline eGFR [1, 15, 17], and baseline CKD class (≥ G3) [1, 3]. Statistical analysis was performed using JMP software version 14.2.0 (SAS Institute, Cary, NC, USA).

## Results

### Patients

**Fig. 3:**
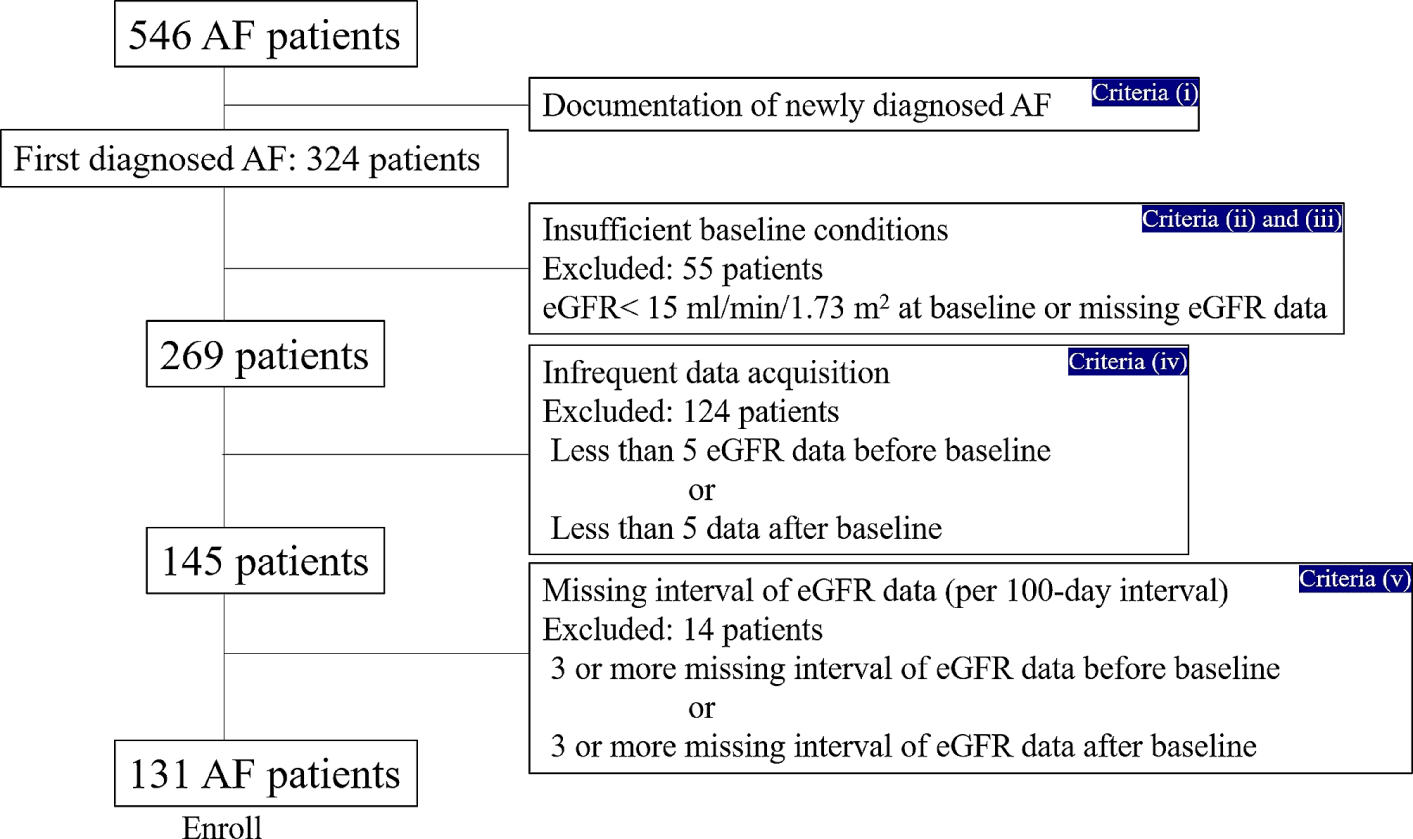
Study population

Initially, 222 patients were excluded for criteria (i). 56 were excluded for criteria (ii) or (iii). 124 patients were excluded for criteria (iv). 14 patients were excluded for criteria (v). Finally, 131 patients were enrolled (Fig. 3).

### Clinical characteristics

The characteristics of the 131 patients are shown in Table 1.

**Table 1:** Clinical characteristics at the first visit for AF

Clinical variables	All patients N = 131
Age (years)	72.3 ± 8.9
Male gender	83 (63.3%)
Body weight (Kg)	61.2 ± 12.8
CHF	51 (38.9%)
Hypertension	92 (70.2%)
Age (> 75 years)	57 (43.5%)
Diabetes mellitus	59 (45.0%)
Stroke	19 (14.5%)
CHADS_2_ score	
0	11 (8.4%)
1	29 (22.1%)
2	39 (29.8%)
3	28 (21.4%)
4	15 (11.5%)
5	8 (6.1%)
6	1 (0.8%)
Vascular disease	31 (23.7%)
Persistent AF	62 (47.3%)
ACE-Is/ARBs	55 (42.0%)
Diuretics	40 (30.5%)
LAD (mm)	43.2 ± 7.0
LVEF (%)	64.1 ± 12.0
Baseline eGFR (mL/min/1.73 m^2^)	62.5 ± 18.4
Baseline CKD class	
G1	8 (6.1%)
G2	65 (49.6%)
G3a and 3b	55 (42.0%)
G4	3(2.3%)

Data are presented as means ± standard deviations or n (%)

*CHF* chronic heart failure, *LAD* left atrial dimension, *LVEF* left ventricular ejection fraction, *ACE-Is*, angiotensin-converting enzyme inhibitors, *ARBs* angiotensin-receptor blockers. *CKD* chronic kidney disease, *G1* eGFR of > 90 ml/min/1.73m^2^, *G2* eGFR of 60–89 ml/min/1.73m^2^, *G3a and G3b* eGFR of 30–59 ml/min/1.73m^2^, *G4* eGFR of 15–29 ml/min/1.73m^2^

### Anticoagulation therapy

Anticoagulation was initiated in 128 of 131 patients at our hospital. Of these, 125 patients were started on anticoagulation therapy on the day of their first visit due to AF, 2 patients were started 1 month after their first visit, and 1 patient was started 3 months after their first visit. There were 3 cases in which anticoagulation therapy was initiated by the primary care physician prior to referral to our hospital. In the referred cases, their exact date of anticoagulation initiation was not traceable, but we assume that most of them started anticoagulation therapy within 1–2 months of their referral to our hospital, based on the contents of the medical information form. 112 patients were started on anticoagulant therapy with direct oral anticoagulants (apixaban in 14 patients, rivaroxaban in 23 patients, edoxaban in 16 patients and dabigatran in 59 patients) at the first visit for AF. Changes in direct oral anticoagulants during the study period were not followed. 19 patients received warfarin at the first visit and were switched to a direct oral anticoagulant after the second visit. Of these, one patient was switched to apixaban, one to rivaroxaban, three to edoxaban and 14 to dabigatran.

### Available eGFR data

The number of available eGFR data per person before baseline was 12 ± 9 (median: 10; range: 5–62), and that after baseline was 15 ± 11 (median: 13; range: 5–69).

### Results of inflection-point analysis

**Fig. 4:**
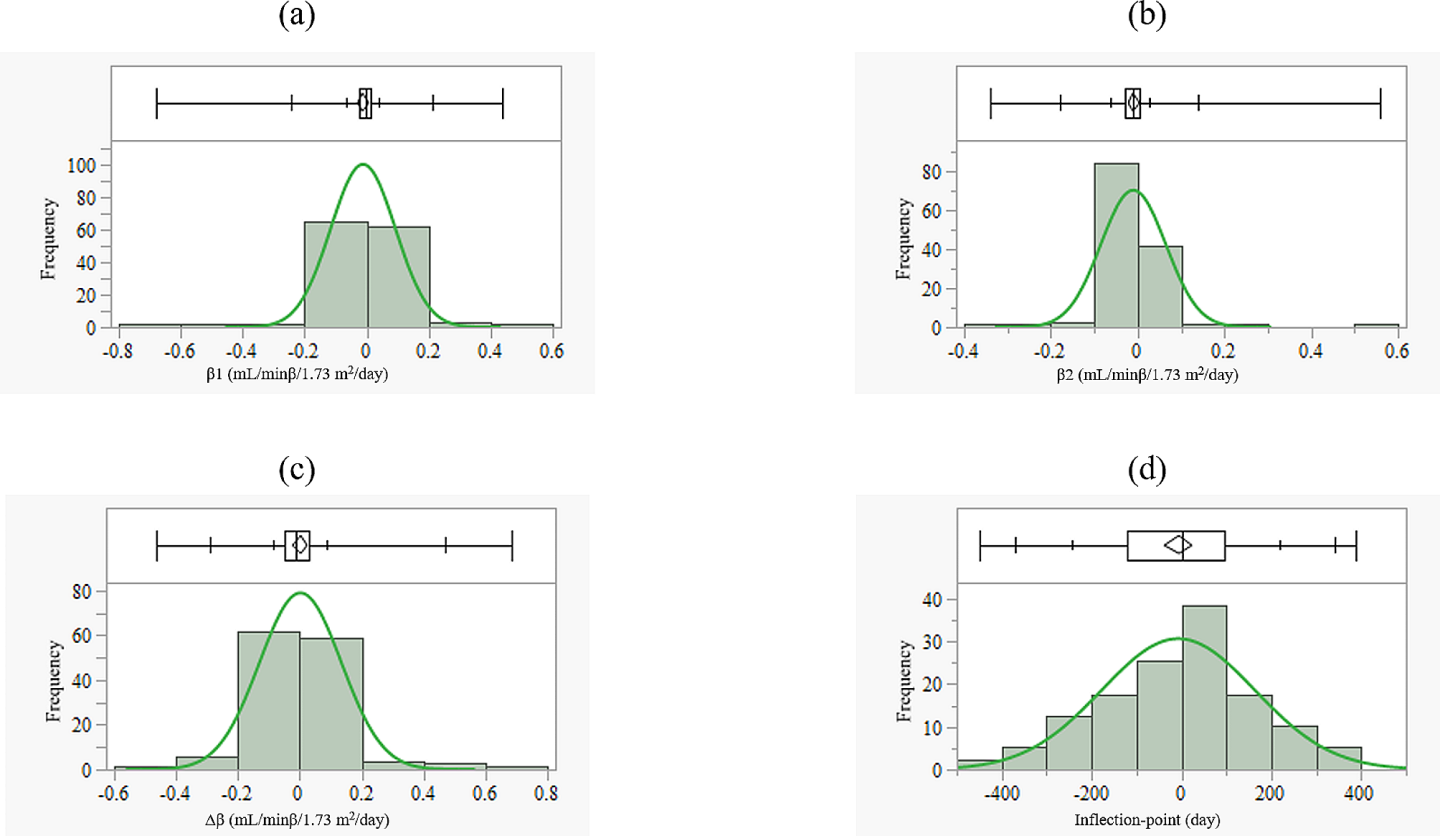
Frequency distribution of β1, β2, Δβ, and inflection-point. A histogram of β1 (**a**). A histogram of β2 (**b**). A histogram of Δβ (**c**). A histogram of the inflection-point (**d**). *β1* slope of the regression line before the inflection-point (units: mL/min/1.73 m^2^/day), *β2* slope of the regression line after the inflection-point (units: mL/min/1.73 m^2^/day), *Δβ* β2 – β1(units: mL/min/1.73 m^2^/day)

Median β1 was − 0.0024 mL/min/1.73 m^2^/day (1st quantile, − 0.0249 mL/min/1.73 m^2^/day; 3rd quantile 0.0126 mL/min/1.73 m^2^/day) (Fig. 4a). The median β2 was − 0.0085 mL/min/1.73 m2/day (1st quantile, − 0.0299 mL/min/1.73 m^2^/day; 3rd quantile 0.0057 mL/min/1.73 m^2^/day) (Fig. 4b). The median Δβ was − 0.0100 mL/min/1.73 m^2^/day (1st quantile, − 0.0871 mL/min/1.73 m^2^/day; 3rd quantile 0.0312 mL/min/1.73 m^2^/day) (Fig. 4c). The median inflection-point was day 0 (1st quantile, day − 120; 3rd quantile day 96) (Fig. 4d).

### Inflection-point with WRF

**Fig. 5:**
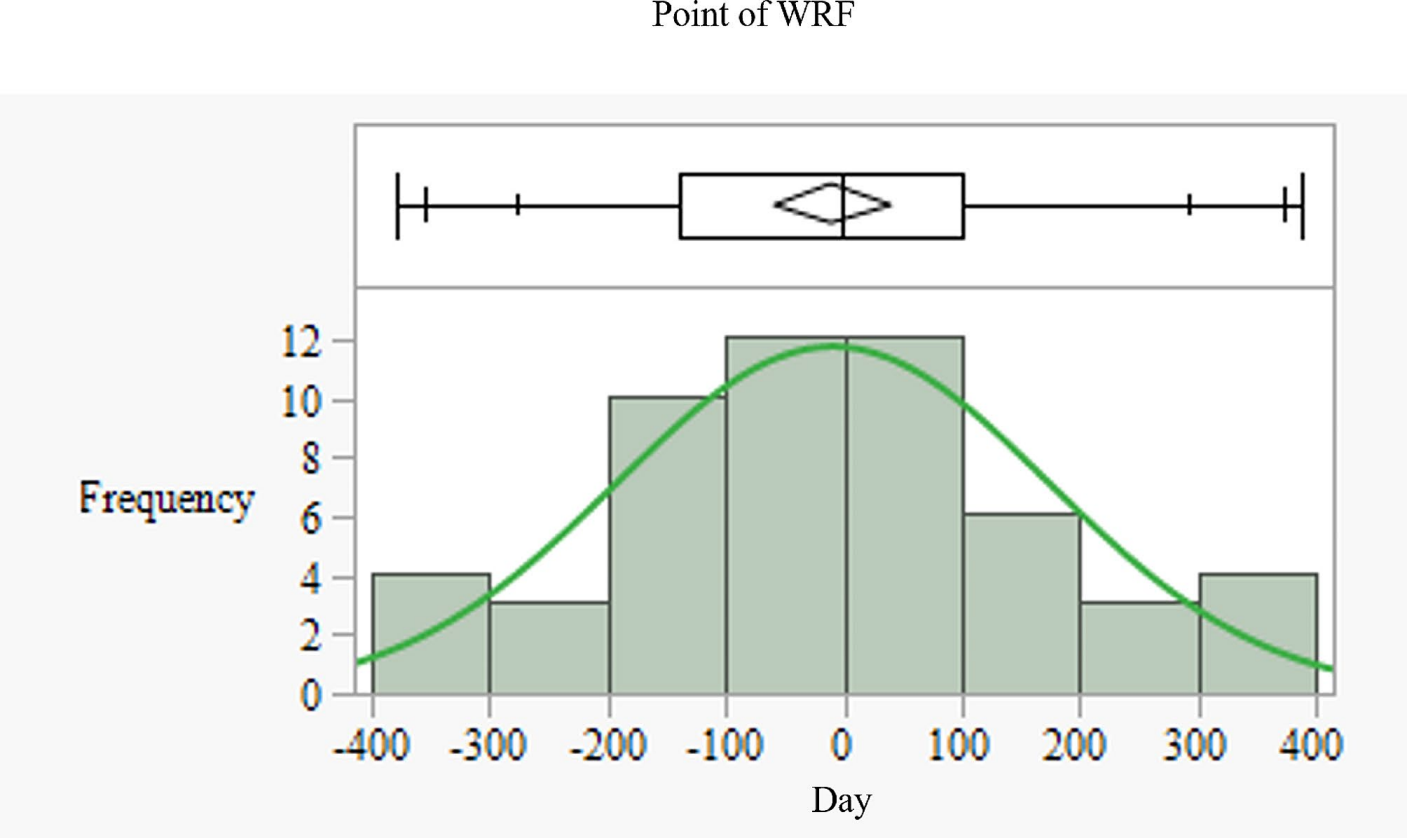
Frequency distribution in the inflection-point with WRF. Box plot indicates median of day − 2, 1st quantile of day − 139, and 3rd quantile of day 100. Green curve indicates the normal distribution fit. *WRF* worsening renal function

**Fig. 6:**
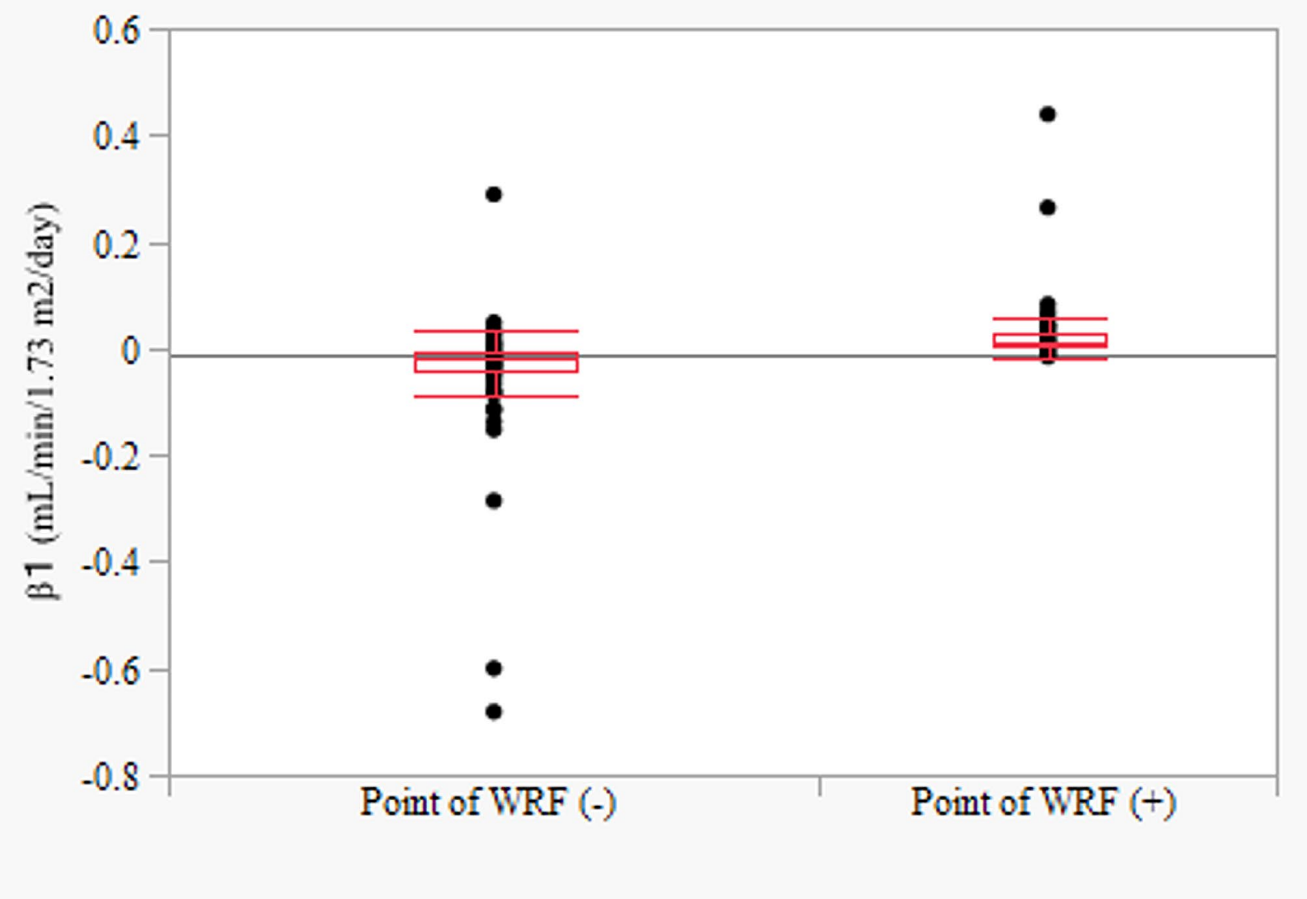
Bivariate relationships between the inflection-point with WRF and β1. *WRF* worsening renal function, *β1* slope of the regression line before the inflection-point (units: mL/min/1.73 m^2^/day)

Inflection-point with WRF was detected in 54 (41.2%) patients. The inflection-point with WRF (i.e., beginning of WRF) was distributed at various times (median day − 2; 1st quantile, day − 139; 3rd quantile day 100), but most frequently (23 out of 54 patients) within 100 days before and after baseline. (Fig. 5). Patients with WRF had significantly higher β1 levels than those without (0.029 ± 0.0134 mL/min/1.73 m^2^/day vs. − 0.041 ± 0.0112 mL/min/1.73 m^2^/day, *P* < 0.01).(Fig. 6). During the study period, patients with WRF had an initial eGFR of 66.4 ± 18.8 mL/min/1.73 m^2^, which decreased to 59.2 ± 18.5 mL/min/1.73 m^2^ by the end of the study. This change was statistically significant.

### Factors associated with WRF

Positive β1 was significantly associated with the presence of WRF (odds ratio 30.5, 95% confidence interval 11.1—83.9, *P* < 0.01). Even after adjustment in a model including all clinical variables investigated at the first visit for AF, a positive β1 was associated with the presence of WRF (odds ratio 51.5, 95% confidence interval 14.6-181.9, *P* < 0.01). (Table 2).

**Table 2:** Multivariate analysis of clinical variables and WRF

Clinical variables	WRF (-) N = 77	WRF (+) N = 54	odds ratio	95% confidence interval	*P* value
Positive β1	16 (20.1%)	48 (88.9%)	51.70	14.66, 182.36	< 0.01
Age (years)	72.4 ± 9.4	72.2 ± 8.4	0.97	0.88, 1.07	0.55
Male gender	48 (62.3%)	35 (64.8%)	1.10	0.28,4.37	0.89
Body weight (Kg)	61.0 ± 13.4	61.4 ± 12.1	1.01	0.96,1.06	0.95
CHF	32 (41.6%)	19 (35.2%)	1.11	0.29,4.26	0.88
Hypertension	50 (64.9%)	42 (77.8%)	7.39	1.09,50.01	0.04
Age (> 75 years)	33 (42.9%)	24 (44.4%)	1.45	0.29,7.12	0.65
Diabetes mellitus	31 (40.3%)	28 (51.9%)	1.32	0.37,4.71	0.67
Stroke	10 (13.0%)	9 (16.7%)	2.16	0.41,11.48	0.36
CHADS_2_ score ≥ 2	49 (63.6%)	42 (77.8%)	0.59	0.08,4.48	0.61
Vascular disease	18 (23.4%)	13 (24.1%)	0.95	0.21,4.39	0.95
Persistent AF	37 (48.1%)	25 (46.3%)	1.12	0.32,3.86	0.86
ACE-Is/ARBs	34 (44.2%)	21 (38.9%)	0.47	0.13,1.72	0.25
Diuretics	26 (33.8%)	14 (25.9%)	0.47	0.12,1.82	0.28
LAD (mm)	43.5 ± 7.7	42.9 ± 5.7	0.97	0.89,1.06	0.56
LVEF (%)	63.4 ± 12.3	65.2 ± 11.6	1.00	0.95,1.05	0.93
Baseline eGFR (mL/min/1.73 m^2^)	62.5 ± 18.4	62.5 ± 18.4	1.01	0.96,1.06	0.74
Baseline CKD class					
G3a, G3b, and G4	38 (49.4%)	20 (37.0%)	0.79	0.15,4.25	0.78
G1	4 (5.2%)	4 (7.4%)			
G2	35 (45.5%)	30 (55.6%)			
G3a and G3b	36 (46.8%)	19 (35.2%)			
G4	2 (2.6%)	1 (1.9%)			

Data are mean ± standard deviation or n (%)

Abbreviations: CHF, chronic heart failure; LAD, left atrial dimension; LVEF, left ventricular ejection fraction; ACE-Is, angiotensin-converting enzyme inhibitors; ARBs, angiotensin-receptor blockers

## Discussion

The main finding of this study is that in almost half of the AF patients who developed WRF, WRF begins in the 100 days before and after the first visit for AF. Although the association between AF and WRF has been shown in several papers, there are no data that use time series eGFR data to estimate the time point at which WRF begins and examine the temporal association with the onset of AF. Furthermore, our findings indicated that an increase in eGFR slope prior to WRF was associated with a subsequent rapid decline in eGFR slope. This finding suggests that the presence of potential renal impairment is a condition that predisposes to the development of cardiorenal syndrome in patients with AF.

### Mechanism by which AF precedes WRF

Our findings indicated that in almost half of the AF patients who developed WRF, WRF began in early onset of AF. Renal hypoxia can be a powerful explanation for the effect of AF on renal function. In an animal model, Freidman et al. reported that AF produced a decrease in cardiac output, leading to decreased blood flow in the renal cortex [18]. Pruijm et al. demonstrated that reduced renal cortical oxygenation predicted subsequent eGFR decline by the blood oxygenation level-dependent magnetic resonance imaging [19]. It is known that in response to renal hypoperfusion, the tubuloglomerular feedback will in turn activate to reduce GFR, resulting in a decrease the metabolic demand and the elimination of oxygen deprivation [20]. And this feedback system breaks down due to continued renal hypoxia, leading to irreversible kidney damage [21, 22]. WRF might be more commonly observed in persistent AF than paroxysmal AF, since persistent AF is exposed to hemodynamics changes due to AF longer than paroxysmal AF. However, a previous report indicated that hospitalization due to heart failure is more common in patients with paroxysmal AF than persistent AF [23]. In patients with paroxysmal AF, acute and repeated changes in heart rate and blood pressure during each AF episode may provide adverse effect on renal function or may not provide enough time to adapt AF hemodynamics. Therefore, we consider that the hemodynamic changes resulting in WRF were not always greater in persistent AF than in paroxysmal AF. GFR is essentially regulated by autoregulation of renal blood flow, however, Hill et al. demonstrated the morphological correlates of loss of autoregulation in the aging kidney of humans [24]. Our findings indicate that AF patients with positive β1 are more susceptible to subsequent adverse effects on renal function, and suggest that positive β1 may represent nephrons overload. Excessive elevation of eGFR is a marker of early renal damage called “hyperfiltration,” which is known to lead to exhaustion of the remaining nephrons and CKD progression [25]. Although eGFR slope was not included as known criteria for hyperfiltration, we believe that positive β1 may indicate hyperfiltration. This is because the kidney undergoes a number of morphological changes with age, as well as a natural gradual decline in clearance function [26, 27]. Therefore, we speculate that a positive β1 against age-related eGFR decline may indicate nephrons overload, and nephrons overload preceding the development of AF may cause exhaustion of the nephrons, resulting in an early eGFR decline after the development of AF. We speculated that the elderly AF patients included those with loss of autoregulation of renal perfusion and glomerular overload, and that the nephrons in these patients were susceptible to the hemodynamic effects of AF, resulting in the onset of WRF early after the development of AF.

### Clinical implications

Kidney management should be initiated at the time of first visit for AF, even in patients with normal renal function. For proper management of direct oral anticoagulation, renal function should be reconfirmed within the first few months after the first visit for AF.

### Limitations

This study has the following limitations. First, because this study was a retrospective, single-center study, the frequency and the interval of available eGFR data were different for each patient. Second, we could not investigate data on proteinuria, which is a known risk factor for CKD progression [28]. Thirdly, we could not investigate the type, dosage, and duration of diuretic medication. In addition, changes in drug therapy including diuretics and ACE-Is/ARB may affect renal function, but this study does not present follow-up data on drug cessation or addition. Fourth, we could not analyze the effect of AF treatment on renal function. The implications of the improvement in eGFR slope are not clear in this study. Treatment of AF and its comorbidities, such as heart failure, may have had a positive impact on renal function. Finally, because it was difficult to determine the date of the first AF episode, the date of the first AF diagnosis was set as the baseline. Patients who had subclinical AF more than a few months before the first visit for AF due to mild subjective symptoms may have been included in this study.

## Conclusion

In nearly half of AF patients with WRF, the beginning of WRF was observed within a few months before or after the first visit for AF. Patients with a positive eGFR slope before the onset of AF are more likely to develop WRF after the onset of AF, suggesting that potential kidney damage may be underlying.

## Data Availability

Data are available upon request to the corresponding author.
